# The emerging role of ectodermal neural cortex 1 in cancer

**DOI:** 10.1038/s41598-023-50914-7

**Published:** 2024-01-04

**Authors:** Lingling He, Chiyu Zhang, Wenjing He, Minjuan Xu

**Affiliations:** 1 Department of Obstetrics, Jiangxi Provincial Maternal and Child Health Hospital, No. 318, Bayi Avenue, Nanchang, 330006 Jiangxi Province China; 2 Department of Urology, The Second Affiliated Hospital of Nanchang University, Nanchang, 330006 Jiangxi Province China; 3 Department of Endocrinology, Baoji Gaoxin Hospital, Baoji, 721006 Shanxi Province China; 4 Department of Obstetrics and Gynecology, Ganzhou People’s Hospital, Ganzhou, 341000 Jiangxi Province China

**Keywords:** Cancer, Biomarkers

## Abstract

Ectodermal neural cortex 1 (ENC1) is a protein that plays a crucial role in the regulation of various cellular processes such as cell proliferation, differentiation, and apoptosis. Numerous studies have shown that ENC1 is overexpressed in various types of cancers, including breast, lung, pancreatic, and colorectal cancer, and its upregulation is correlated with a poorer prognosis. In addition to its role in cancer growth and spreading, ENC1 has also been linked to neuronal process development and neural crest cell differentiation. In this review, we provide an overview of the current knowledge on the relationship between ENC1 and cancer. We discuss the molecular mechanisms by which ENC1 contributes to tumorigenesis, including its involvement in multiple oncogenic signaling pathways. We also summarize the potential of targeting ENC1 for cancer therapy, as its inhibition has been shown to significantly reduce cancer cell invasion, growth, and metastasis. Finally, we highlight the remaining gaps in our understanding of ENC1’s role in cancer and propose potential directions for future research.

## Introduction

Cancer is a complex disease that poses significant challenges to patient survival. To diagnose and treat cancer effectively, identifying molecular markers that play a role in both diagnosis and prognosis is critical. One such marker is Ectodermal neural cortex 1 (ENC1), a gene linked to kelch, an important gene for oogenesis in Drosophila^1^. ENC1 has been shown to play a role in neural differentiation, with its expression pattern throughout development and in vitro studies indicating increased expression during neuroblastoma cell neuronal differentiation. In humans, ENC1 is highly expressed in the brain and spinal cord, and its gene is located on chromosome 5q13^2^. ENC1, also referred to as NRP/B (nuclear restricted protein/brain), serves as a nuclear matrix protein, demonstrating up-regulation during neuronal differentiation. Its significance in the differentiation process lies in its interaction with the hypophosphorylated form of the retinoblastoma protein (p110RB). NRP/B plays a role in cell cycle withdrawal post-commitment to differentiation. Alternatively, it may influence the regulation of neuronal cell differentiation by disrupting the function of cell cycle regulatory proteins, including p110^RB^^3^.

ENC1 is also involved in the development of adipocytes and neural crest cells, and its dysregulation has been linked to necrotizing gastroenteritis and neuroblastoma^2,4^. The gene is associated with the nuclear receptor meta-pathway and the glucocorticoid receptor pathway, and its function includes actin binding and control of mutant neurotoxicity Huntingtin aggregation through p62 during endoplasmic reticulum stress. Additionally, ENC1 regulates the activity of the transcription factor Nrf2 in defense against oxidative stress, and there is evidence to suggest that its expression contributes to carcinogenesis^5^.

This article focuses on the relevance of ENC1 in cancer and the potential impact of targeting ENC1 in cancer treatment. By understanding the role of ENC1 in cancer development and progression, we can explore new therapeutic approaches that may ultimately improve patient outcomes.

## ENC1, a gene that promotes cancer

**Figure 1:**
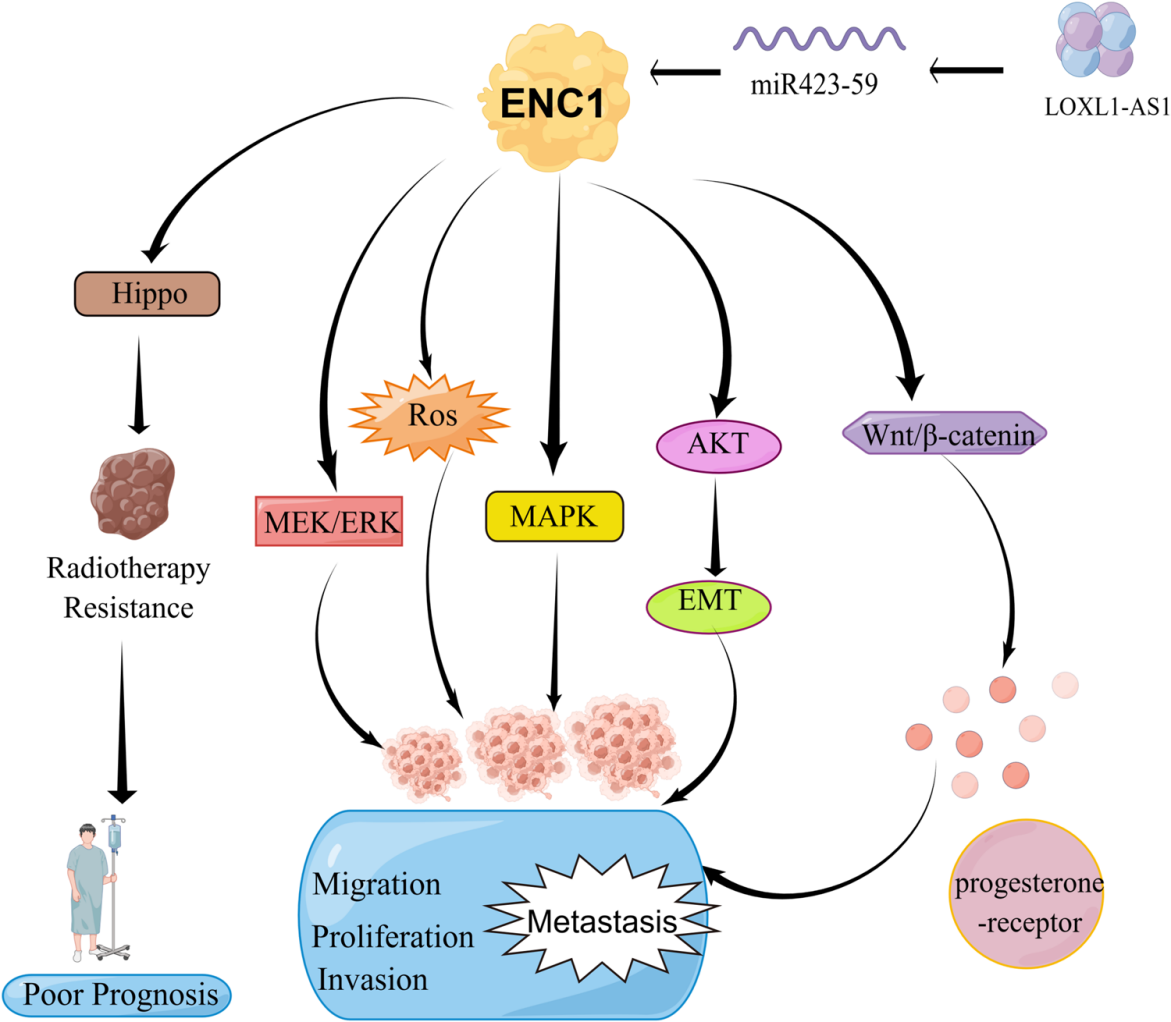
Mechanistic map illustrating the role of ENC1 in promoting tumor progression through multiple signaling pathways and molecules. Hippo, Hippo signaling pathway; ENC1, Ectodermal neural cortex 1; MEK/ERK, MEK/ERK signaling pathway; ROS, Reactive oxygen species; MAPK, MAPK signaling pathway; AKT, Protein kinase B; EMT, Epithelial-mesenchymal transition; Wnt/β-catenin, Wnt/β-catenin pathway; LOXL1-AS1, Long non-coding RNA LOXL1 antisense RNA 1.

The ENC1 protein is known to possess a complex BTB domain-like structure in its NH2 terminus and a “kelch motif” in its COOH-terminal region, as revealed by recent studies^3^. In malignancies, ENC1 has emerged as a key player (Fig. 1). Upregulation of ENC1 has been observed in various cancers, including breast, colon, lung, and ovarian cancer^6–10^. Studies suggest that ENC1 may modulate tumor cell malignancy through reactive oxygen species and various signaling pathways. Additionally, ENC1 has been found to impact the radiosensitivity of super-enhancer-driver-driven tumor cells, which can have an impact on cancer patient prognosis^7^. Given these findings, ENC1 is being explored as a promising new diagnostic marker for cancer.

### ENC1 and ROS

ENC1 has been found to play a role in regulating oxidative stress^11^, which is characterized by the presence of reactive oxygen species (ROS) such as hydrogen peroxide (H_2_O_2_). ROS are generated during normal cellular metabolism and in response to stress. The inhibitory effect of H_2_O_2_ on cell growth has been observed in a dose-dependent manner, indicating that higher concentrations of H_2_O_2_ can cause oxidative stress and damage to cells^12^.

### ENC1 and β-catenin pathway

β-catenin is a multifunctional protein that plays a crucial role in maintaining normal body functions, and its aberrant high expression is associated with various diseases, including cancer. In addition to functioning as a protein involved in intracellular adhesion, β-catenin also acts as a co-regulator of transcription. Wnt is one of the main regulators of β-catenin^13^. The Wnt/β-catenin signaling system is involved in embryogenesis, stem cell renewal, and normal tissue maintenance. However, abnormal activation of this pathway leads to nuclear accumulation of β-catenin and stimulates transcription of several oncogenes, such as c-Myc and cyclin D1^14^. Cancer progression is often associated with aberrant activation of the Wnt/β-catenin signaling pathway. ENC1 has been shown to be closely linked to cancer metastasis by modulating the β-catenin pathway^15^.

### ENC1 and MAPK pathway

The MAPK pathway has diverse substrates and can trigger various responses based on the input signal it receives^16^. Several human diseases, including Parkinson’s disease and various cancers, are associated with the pathophysiology of MAPK signaling pathways^17^. MAPK is composed of four subfamilies: ERK, p-p38, JNK, and ERK5. ERK is present in various mammalian organs and regulates cell growth and differentiation. ERK activation is required to complete the signal transduction cascade for a variety of growth factor and nutrition-related factor receptors^16^. JNK can promote apoptotic cell death, inhibiting tumor growth, or make tumors more aggressive and drug-resistant by promoting cancer stem cell renewal, cell migration, and extended survival of cancer cells^16^. The p38 pathway is usually activated in response to environmental stress and inflammation and plays a crucial role in maintaining cellular homeostasis in various tissues, including the nervous and cardiovascular systems, and in cancer. In the presence of an oncogene, reactive oxygen species (ROS) are generated, triggering p38-mediated proapoptotic signaling and eliminating early tumor cells^18^. Early in tumor progression, p38 activation often suppresses tumor growth, but once a tumor has formed, it promotes its development^19^. Knockdown of ENC1 markedly decreased ERK, JNK, and p38 levels in lung cancer^9^.

### ENC1 and EMT

Epithelial-mesenchymal transition (EMT) is a cellular process whereby epithelial cells undergo a transformation into mesenchymal cells^20^. EMT is associated with various tumor-related activities, such as tumor initiation, malignant progression, stemness, migration, invasion, metastasis, and resistance to treatment^20,21^. EMT is characterized by the loss of the epithelial marker E-cadherin and the acquisition of the mesenchymal marker vimentin^20^. In lung cancer cells transfected with si-ENC1, there was a significant decrease in N-cadherin expression and a marked increase in E-cadherin expression^9^. These findings suggest that ENC1 may play a role in regulating EMT in lung cancer and may be a potential therapeutic target for this disease.

## ENC1 and its role in cancer

ENC1 is highly upregulated during pregnancy, and studies have shown that it is expressed abnormally in both myelodysplastic syndromes and solid tumors^22^. Overexpression of ENC1 has been correlated with the stage of the tumor and has been used as a prognostic marker for disease-free survival and overall survival^10^. In breast cancer, ENC1 overexpression has been linked to increased bone and brain metastases, and may impact the prognosis of cancer by altering tumor cell sensitivity^7^. ENC1 is involved in tumor cell angiogenesis, invasion, and metastasis through matrix metalloproteinases, and encourages tumor metastasis through EMT^9^. In this paper, we review studies on ENC1 in gastrointestinal malignancies, gynecological neoplasms, respiratory system and hematological system tumors.

### ENC1 in gastrointestinal cancers

#### Colorectal cancer collaborators

Colorectal cancer (CRC) is a significant global health threat, being the third most common cancer and second leading cause of cancer-related deaths worldwide according to the Colorectal Cancer Collaborators^23^. Overexpression of ENC1 has been linked to poor patient survival in CRC, with higher expression levels correlating with advanced T stage and unfavorable clinical outcomes. Studies have shown that ENC1 promotes the growth and metastasis of CRC through the JAK2-STAT5-AKT signaling axis. Knockdown of ENC1 in mouse xenotransplantation and lung metastasis models inhibits tumor growth and metastasis^8^. Moreover, ENC1 is regulated by the β-catenin/TCF system, and its altered expression may contribute to colorectal carcinogenesis by inhibiting colonic cell differentiation^24^. Thus, ENC1 may serve as a diagnostic marker for CRC and targeting ENC1 may hold potential as a therapeutic strategy to combat CRC.

### ENC1 in gynecological neoplasms

#### Ovarian cancer

Ovarian cancer is one of the most common and deadliest forms of cancer in women. Epithelial ovarian cancer, the most prevalent type, originates from the epithelium lining the fallopian tubes and is often diagnosed at an advanced stage, making it the leading cause of death from gynecological malignancies^25^. To improve survival in this aggressive disease, extensive molecular and cellular profiling is being undertaken, recognizing the challenges associated with its treatment. Access to evidence-based treatment is essential for enhancing survival in this aggressive disease. Precision medicine involves prioritizing clinical trials of novel therapeutics and developing predictive biomarkers to select patients who will benefit from chemotherapy, targeted agents, and immunotherapy. Low ENC1 expression has been linked to longer DFS and OS in ovarian cancer patients, and studies have shown that ENC1 expression is associated with FIGO stage in ovarian cancer^10^. Downregulation of ENC1 expression in ovarian cancer cells may lead to increased ROS production, which can cause oxidative stress, cellular damage, and dose-dependent growth inhibition^10^. Therefore, ENC1 expression may be a useful predictor of ovarian cancer prognosis.

#### Cervical cancer

Cervical cancer (CC) is the fourth most prevalent illness among women and the fourth leading cause of cancer-related death^26^. Chronic HPV infection is responsible for approximately 91% of cervical cancer cases^27^ For the treatment of advanced gynecological cancers, radical hysterectomy or terminal chemotherapy is generally recommended. The co-administration of radiation and systemic cisplatin-based chemotherapy has been found to improve local control and overall survival in randomized clinical trials when compared to radiation alone^28^.

Numerous studies have shown that dysregulation of long non-coding RNAs (lncRNAs) actively contributes to CC progression. LOXL1-antisense RNA 1 (LOXL1-AS1) has been identified as a pro-cancer regulator in various malignancies, and its upregulation is strongly associated with a poor prognosis for cervical cancer^29^. In vivo studies have shown that inhibiting LOXL1-AS1 expression reduces Ki67 expression and prevents tumor development and spread. Similarly, miR-423-5p has been shown to suppress the growth and invasion of osteosarcoma, ovarian cancer, and prostate cancer tumor cells^29–31^, and has an antitumor effect in cervical cancer cells as well.

The MAPK/ERK pathway controls numerous physiological and pathological events throughout cancer development. Knockdown of ENC1 has been shown to reduce the levels of the proteins p-p38, p-MEK1/2, and p-ERK1/2, which limits the activation of the ERK/MEK pathway and slows cell growth^29^. However, the overexpression of ENC1 may somewhat counteract the downregulation of cellular phenotypes by miR-423-5p overexpression in CC^29^. Taken together, the LOXL1-AS1/miR-423-5p/ENC1 axis accelerates CC production through the MEK/ERK pathway^29^.

#### Endometrial carcinoma

Endometrial carcinoma (EC) is a prevalent gynecologic cancer with an increasing global incidence. The rising occurrence of EC poses a serious threat to women’s health due to shifting lifestyles and the widespread use of estrogen replacement therapy^32^. Late diagnosis is a major contributor to EC-related deaths. Tumor cells in EC interact with the host stroma, which consists of immune cells, endothelial cells, fibroblasts, and metabolites, in the tumor microenvironment (TME). ENC1 mRNA and protein expression levels are overexpressed in EC tumor tissues and cell lines^33^. High expression of ENC1 in endometrial carcinoma suggests poor prognosis. However, the molecular mechanisms by which ENC1 promotes EC progression and its role in pathway communication remain unclear.

ENC1 expression has been shown to be associated with CD8+ T cells and neutrophils but not with CD4+ T cells or B cells^33^. This suggests that immune cell infiltration in patients with EC may be related to the level of ENC1 expression, but further research is needed to validate this hypothesis. Immunotherapy, which currently plays a significant role in cancer therapy, involves blocking immunological checkpoints. ENC1 is negatively correlated with CD244 and CTLA4 but positively correlated with other immunological checkpoints (CD274 and CD276). Future immunotherapy against EC may target ENC1^34^. In summary, ENC1 is a potential therapeutic target in endometrial carcinoma, and further research is needed to understand its precise role in promoting cancer progression and its interaction with the immune system in the TME.

#### Breast cancer

Breast cancer is the leading cause of cancer-related deaths in females globally as of 2020^26^. Approximately 2.3 million women received a diagnosis of breast cancer that year, and 685,000 lost their lives to the disease. While breast cancer can occur at any age, the incidence rate increases with age. Breast cancer tissue has been found to express higher levels of ENC1 than normal breast tissue, which may enhance the capacity of breast cancer cells to proliferate and spread^15^. Increased ENC1 expression is a potential diagnostic indicator in individuals with breast cancer and is associated with a poor prognosis. ENC1 has been linked to cell contact and cell adhesion in breast tissue, and analysis of TCGA and GEPIA datasets revealed that ENC1 and β-catenin expression are strongly correlated with breast cancer^15^. Thus, ENC1 may potentially contribute to the development of breast cancer through the Wnt/β-catenin signaling pathway. Inhibiting ENC1 in breast cancer cells can inhibit cell proliferation, migration, invasion, and colony formation. Additionally, the fact that high levels of ENC1 expression are predictive of low levels of PR expression suggests the existence of a potential negative feedback loop between the β-catenin pathway and the progesterone steroid receptor, with ENC1 acting as an inhibitor of the progesterone steroid receptor through the β-catenin pathway^15^.

A meta-analysis has shown that ENC1 is overexpressed in breast cancer (BC) and that this gene is associated with radiation reactions^7^. Inhibition of ENC1 was found to increase the rate of apoptosis in tumor tissues following radiation treatment. These findings suggest that inhibiting ENC1 can reduce the malignant biological activity of BC cells and increase their radiosensitivity. Super-enhancers (SE) are groups of transcription enhancers that promote the expression of genes, including oncogenes, that are important for tumor development in various malignancies, such as neuroblastoma, lung cancer, esophageal cancer, and gastric cancer^35,36^. ENC1 is an SE-driven gene that is regulated by transcription factor 4 (TCF4)^35^. Abnormal SE-driven ENC1, regulated by TCF4, has been linked to radio-resistance and a poor prognosis. Activation of the Hippo pathway by ENC1 enhances radio-resistance in BC cells^35^. Specifically, a 10-kb long SE located 60 kb upstream of ENC1 promotes its expression through translationally promoting TCF4. This, in turn, inhibits Last1/2, YAP1, and TAZ phosphorylation of Hippo signaling, leading to an increase in BC cell recurrence and radio-resistance. These findings may help identify a BC radio-resistance biomarker and treatment target.

### Respiratory system cance

#### Lung cancer

Despite recent therapeutic advances, the majority of lung cancer patients are diagnosed at a late stage, which has a detrimental effect on survival. Lung cancer is the second most common and deadly form of cancer worldwide^37^. However, by identifying modifiable risk factors and implementing early screening measures, the incidence of lung cancer may be reduced. ENC1 is highly expressed in lung cancer tissues, and downregulating this gene has been shown to effectively inhibit lung cancer cell proliferation and migration, while altering the expression of other proteins^9^. A nude mouse model of subcutaneous tumor xenotransplantation was developed to investigate the effects of ENC1 downregulation on cancer cells. In contrast to paracancerous tissues and normal lung cells, the expression of ENC1 was found to be significantly higher in lung cancer tissues and cells. Knocking down ENC1 using si-ENC1 gene significantly slowed cell proliferation, migration, and invasion compared to non-transfected cells^9^. In addition, levels of MMP-2, MMP9, N-cadherin, JNK, ERK, and p-p38 all decreased when ENC1 was turned off, indicating that si-ENC1 blocks the MAPK pathway, thereby preventing lung cancer cells from proliferating, migrating, and invading^9^. In summary, ENC1 is greatly overexpressed in lung cancer and may be used as a diagnostic and prognostic marker^9^. Moreover, targeting ENC1 could be a potential strategy for the diagnosis and treatment of malignancies other than non-small cell lung cancer.

### Hematological system tumor

#### Myelodysplastic syndrome

Myelodysplastic syndrome (MDS) is a clonal hematopoietic disorder that predominantly affects older individuals and is associated with an increased risk of developing acute myeloid leukemia (AML) and bone marrow failure. MDS is characterized by multilineage cytopenia, ineffective hematopoiesis, and increased production of blasts in the bone marrow^38,39^. The stromal cell compartment, which includes mesenchymal stromal cells (MSCs), plays a crucial role in the pathophysiology of MDS by inducing proinflammatory cytokines and hypoxia, which disrupt the hematopoietic niche and promote disease progression^40^. In addition, MDS patients have increased numbers of myeloid-derived suppressor cells (MDSCs) in their tumor microenvironment, which are generated by the conversion of healthy monocytes into MDSCs by MDS-MSCs and can impair the immune response^40^. Research has shown that the expression of ENC1 is upregulated in MDS-MSCs, and this upregulation is induced by TGF-β in monocytes. ENC1, a regulator of cellular oxidative stress, promotes the generation of reactive oxygen species (ROS) by inhibiting Nrf2^41,42^. In turn, the increased ROS levels and other changes in monocytes induced by ENC1 contribute to the immunosuppressive environment in MDS and promote tumor development^22^. Knocking down ENC1 in MDS-MSCs leads to a reduction in TGF-β in co-cultured monocytes and the elimination of immunosuppression^22^. Taken together, these findings suggest that targeting ENC1 may represent a promising strategy for improving immune function and preventing disease progression in MDS.

#### Hairy cell leukemia

Hairy cell leukemia (HCL) is an uncommon chronic lymphoid leukemia that originates from mature B lymphocytes^43,44^. The frequency of HCL is 0.3 instances per 100,000 individuals, with a median age at diagnosis of 58 years^45^. In the US, around 1000 new HCL cases are diagnosed annually, with a four-fold higher incidence in males compared to females^46^]. Karyotypic alterations of chromosome 5, particularly band 5q13, are frequently observed in HCL patients^47^. Notably, several genes have been identified in close proximity to a constitutional inv^5^ (p13.1q13.3) breakpoint in one HCL patient, including beta-hexosaminidase HEXB, a gene commonly mutated in Sandhoff disease, a lysosomal storage illness. A unique alternate isoform of HEXB disrupted by the 5q13.3 breakpoint has been designated as ENC-1AS, which overlaps directly with exon 1 of the gene for ectodermal neuronal cortex 1 in a cis-antisense manner. ENC1 expression is evident in hairy cell leukemia patients, and ENC-1AS regulates ENC1 at the RNA sequence level, rather than via its protein-coding potential ^47^.

HCL cells express both CD27 and its ligand CD70, which are believed to be essential for plasma cell development from memory B-cells^48,49^. This finding suggests a possible mechanism for ENC1 expression and a role for ENC1 in B cell differentiation. Furthermore, F-actin, a vital component of protrusions on hairy cells’ surface, is known to interact with ENC1, a protein that binds to F-actin^50^. Thus, upregulation of ENC1 may contribute to HCL development, and the emergence of ENC-1AS, a novel antisense gene, may indicate dysregulation of ENC1. To better understand the involvement of ENC1 in HCL development and other neoplasms, further studies are necessary to elucidate the regulatory mechanisms of ENC1 expression and the actions of the ENC1 protein.

## ENC1 as a therapeutic target in cancer

Numerous studies have implicated ENC1 in the pathogenesis of various malignancies. In cancer patients, high levels of ENC1 expression have been linked to increased malignancy, metastasis, and a poorer prognosis. Conversely, reducing ENC1 expression via knockout has been shown to significantly inhibit cancer cell invasion, growth, and metastasis, and improve patient prognosis. Several mechanisms have been explored to target ENC1, including the ROS, β-catenin, MAPK, and EMT pathways. Given the ability of targeted ENC1 to inhibit cancer cell growth, induce apoptosis, and modulate radiotherapy sensitivity, it may represent a promising new therapeutic strategy for cancer treatment. However, there are currently no clinical trials or studies focused on targeting ENC1 for cancer treatment. Further research is warranted to fully elucidate the therapeutic potential of targeting ENC1 in cancer.

## Future perspective

As our understanding of the complex interactions between oncogenic pathways and cellular activities in different malignancies continues to grow, it is becoming increasingly clear that the key to developing more effective cancer therapies lies in understanding how genetic alterations activate tumor growth and signaling pathways. Future research will likely focus on identifying specific genetic mutations and cellular processes that contribute to cancer development, as well as developing targeted therapies that exploit these vulnerabilities. Additionally, emerging technologies such as gene editing and immunotherapy hold great promise for improving cancer treatment outcomes. With continued advances in cancer research and therapy, we can hope for a future where cancer is no longer a devastating disease, but a manageable chronic condition.

## Conclusion

In addition to its effect on the production of the transcription factor NF2L2/NRF2, ENC1-encoded actin has been shown to play a role in controlling neuronal process development and neural crest cell differentiation. Given its versatility, it is possible that ENC1’s impact on cancer growth and metastasis is not limited to a single substrate, but rather affects multiple signaling pathways simultaneously. To gain a better understanding of how ENC1 affects cell survival and homeostasis, further investigation is needed to explore its relationship with cancer-related signaling pathways, potential targets, regulatory mechanisms that govern its expression, and molecular abnormalities common to various cancer types. Collectively, the evidence suggests that ENC1 may be a novel target for the development of new therapeutic options for cancer treatment.

## Data Availability

The datasets used and/or analyzed during the current study are available from the corresponding author upon reasonable request.
